# A Prescription for Resistance: Management of Staphylococcal Skin Abscesses by General Practitioners in Australia

**DOI:** 10.3389/fmicb.2016.00802

**Published:** 2016-06-06

**Authors:** Christine Parrott, Gillian Wood, Ekaterina Bogatyreva, Geoffrey W. Coombs, Paul D. R. Johnson, Catherine M. Bennett

**Affiliations:** ^1^Centre for Population Health Research, School of Health and Social Development, Deakin University, GeelongVIC, Australia; ^2^Young People’s Health Service, Department of Adolescent Medicine, The Royal Children’s Hospital, ParkvilleVIC, Australia; ^3^Perinatal Medicine, Mercy Hospital for Women, HeidelbergVIC, Australia; ^4^Department of Microbiology, Dorevitch Pathology, HeidelbergVIC, Australia; ^5^School of Veterinary and Life Sciences, Murdoch University, MurdochWA, Australia; ^6^PathWest Laboratory Medicine – Fiona Stanley Hospital, MurdochWA, Australia; ^7^Department of Infectious Diseases, Austin Health, HeidelbergVIC, Australia

**Keywords:** antibiotic resistance, *Staphylococcus aureus*, skin and soft tissue infections, community medicine, boils and abscesses

## Abstract

**Objectives:** We investigated the management of staphylococcal abscesses (boils) by general practitioners (GPs) in the context of rising antibiotic resistance in community strains of *Staphylococcus aureus*.

**Design, Setting, Participants:** We analyzed patient-reported management of 66 cases of uncomplicated skin abscesses from the frequency matched methicillin-resistant *S. aureus* (MRSA) and methicillin-sensitive *S. aureus* (MSSA) Community-Onset *Staphylococcus aureus* Household Cohort (COSAHC) study (Melbourne, Australia, 2008–2012). Susceptibilities in all cases were known: 50/66 abscesses were caused by MRSA. In order to investigate GP-reported management of staphylococcal abscesses, we surveyed a random subset of GPs, from the COSAHC study (41), and of GPs (39) who used the same community-based pathology service (December 2011–May 2012).

**Main outcome measures:** Patient outcomes, antibiotics prescribed, antibiotic resistance profiles of infecting strains, rates of incision and drainage (I&D), and attitudes to ordering microbiological cultures.

**Results:** MRSA was three times more likely to be cultured from an abscess than MSSA. Patient-reported management revealed 100% were prescribed antibiotics and only 60.6% had I&D. Of those 85% who remembered their prescription(s), 81% of MRSA cases and 23% of MSSA cases initially received inactive antibiotics. Repeat GP visits where antibiotics were changed occurred in 45 MRSA and 7 MSSA cases, although at least 33% of subsequent prescriptions were inactive for the MRSA infections.

Patients treated with I&D and antibiotics did no better than those treated with only I&D, regardless of the antibiotic activity. In the GP surveys, 89% reported I&D, with or without antibiotics, to be their preferred management. Only 29.9% of GPs would routinely swab abscesses.

**Conclusion:** The recommended management of uncomplicated *Staphylococcus* abscesses is I&D without antibiotics to reduce exposure to unnecessary antibiotics. In our study, I&D was performed in only 60.6% of 66 patients, and antibiotics were always prescribed. The prescribed antibiotics were frequently inactive and often changed, and did not appear to affect patient recovery. Our results show that community GPs can confidently reduce their use of antibiotics for patients with skin abscesses and should be aware that MRSA is a much more common in this type of infection.

## Introduction

Little is known about Australian general practitioners’ (GPs) management protocols in practice for skin abscesses and how this might be evolving with the changing epidemiology of *Staphylococcus aureus*. Australian Therapeutic Guidelines: Antibiotic (version 15; Antibiotic Expert Groups, 2014) recommends *uncomplicated* abscesses should be managed with incision and drainage (I&D) alone – without antibiotics. Previous guidelines (Dermatology Expert Groups, 2009) available to GPs at the time of our study, also recommended only I&D for abscesses <5 cm. Antibiotics were only recommended if there was associated spreading cellulitis or systemic symptoms.

After a 2011 review, clinical practice guidelines from the Infectious Diseases Society of America (IDSA) continued to recommend I&D; specifically, that effective treatment requires incision, thorough evacuation of pus, and probing the cavity to break up loculations (Liu et al., 2011; Moran et al., 2013). IDSA practice guidelines are similar to the Australian Therapeutic Guidelines for primary treatment (CDC, AMA, IDSA, 2007; Lowy et al., 2008; Liu et al., 2011; Centers for Disease Control and Prevention [CDC], 2013; Moran et al., 2013). However, they differ in their recommended first line antibiotics in cases where antibiotics are indicated, due to higher rates of community-acquired MRSA (CA-MRSA) in the United States. The IDSA guidelines recommend clindamycin, trimethoprim-sulfamethoxazole, a tetracycline (doxycycline/minocycline) and linezolid (Liu et al., 2011). In contrast, the recommended antibiotics for complicated abscesses in the Australian Therapeutic Guidelines are di/flucloxacillin or, in penicillin-allergic patients, cephalexin. In penicillin hypersensitive patients, the recommendation is to use clindamycin or trimethoprim-sulfamethoxazole. Antibiotics are to be modified based on culture results (Antibiotic Expert Groups, 2014).

We have calculated that in 2006, 3–5% of patients presenting with community-onset *S. aureus* infections in Melbourne had an MRSA infection (Bennett et al., 2014). The preliminary findings of the COSAHC study suggest this has risen to 8–10% by 2010, and may be higher again when aggressive pyogenic soft tissue *S. aureus* infections have been investigated, showing MRSA the more likely causative organism (Jahamy et al., 2008; del Giudice et al., 2009; Coombs et al., 2014). This study will show that CA-MRSA is three times more likely than MSSA to be the cultured organism in skin abscesses.

With CA-MRSA increasing in prevalence in Australia and elsewhere, there may be an argument for all lesions to be swabbed for microbiological culture and sensitivity (MC&S), not only to tailor treatment (as in complicated infections) but also for MRSA surveillance (CDC, AMA, IDSA, 2007; Lowy et al., 2008; Dermatology Expert Groups, 2009; Liu et al., 2011; Centers for Disease Control and Prevention [CDC], 2013; Antibiotic Expert Groups, 2014). However, for uncomplicated abscesses, if, as is recommended, I&D is routinely and correctly used, (thorough evacuation of pus, and probing the cavity to break up loculations) and antibiotics are *not* used, then swabs may have little impact on individual care (Parnes et al., 2011). Community GPs may not realize that CA-MRSA is the most likely organism to cause uncomplicated abscesses. Further over prescription may occur with the return of a culture and sensitivity report, indicating the prescribed (unnecessary) antibiotic is inactive, which may drive an antibiotic change, resulting in even more ineffective antibiotics as well as unnecessary visits to the GP.

Our study set out to describe the management by community GPs of staphylococcal skin abscesses so we can better understand how often I&D is performed, if and what types of antibiotics are used, and whether antibiotics affect patient recovery. We also wanted to understand how often and for what reason GPs send swabs for MC&S.

## Materials and Methods

### Patient Data

Community-Onset *Staphylococcus aureus* Household Cohort is a longitudinal cohort study of 291 index patients with community-onset *S. aureus* infections, and 446 household contacts. Index patients were recruited through a large private pathology provider on the basis of a positive *S. aureus* MC&S result for their infection. The pathology provider serves the entire Melbourne metropolitan area. All eligible patients with community-onset MRSA infections were invited to participate together with a frequency-matched subset of eligible patients with MSSA infections. The COSAHC project was approved by the Deakin University Human Research Ethics Committee (project number 2009-162).

Shared households (204) were followed up at 3-monthly intervals for up to 2 years (recruitment period 2008–2011). At each visit, index patients and household members had swabs obtained from nares and axillae for *S. aureus* carriage to determine the molecular epidemiology of strains circulating within households. Questionnaires were completed to provide information on the medical management and outcome of the index cases’ infection as well as household interactions, infection history, new infections and risks for *S. aureus* transmission and MRSA carriage.

Of the 291 index patients, 137 had MRSA infections and 154 had MSSA infections. The majority were skin and soft tissue infections (86.2%), and 66 of the index patients had skin abscesses (50 MRSA, 16 MSSA) and are included in the present analysis. We extracted data on the doctor-reported data management (when remembered) as well as patient-reported management and outcomes for these 66 skin abscess infections; including whether I&D was performed or if and what type of antibiotics were prescribed. We also established the resistance profile of the infecting strain, and the number of days off normal activities and timing of infection resolution.

### Doctor Survey Data

To understand the clinical decisions behind treatment practices, we conducted a cross-sectional survey of community GPs on their management protocols for *S. aureus* abscesses using a constructed case study (see below). Participating doctors were from one of two groups, recruited by telephone and fax from December 2011 to May 2012. The survey tool asked a series of questions on their treatment of this hypothetical case, and decisions to swab.

**Case Study Extract**

A patient presents to your clinic with a boil in their right armpit. Over the course of 3 days, the area has become increasingly reddened, tender and the center is raised and now forms a pus-filled head. There are no signs of systemic infection but there is evidence of localized infection. The patient has no other health problems and is not on any medication.

1. With this history, how would you treat the patient? (Select 1 or more)
  (a) No treatment
  (b) Incision and drainage (I&D)
  (c) I&D and antibiotics
  (d) Antibiotics only
  (e) Other (please specify)
2. If this boil was weeping or lanced*, would you swab this boil for culture and if so why?

Followed by a series of multiple choice and open ended questions on treatment choices and motivators for, and frequency of, collecting swabs.

^∗^*Lanced* used interchangeably with *incision*

#### Group 1: Doctors Who Ordered the Baseline Tests on Patients Who Were Recruited to the COSAHC Study

We surveyed a random sample of 41 doctors treating community-onset *S. aureus* infections that had previously been studied in COSAHC. A subset of these doctors had treated eight COSAHC- patients who had an abscess as their index infection, allowing us the additional opportunity to directly compare GP-reported management protocols from the survey to the real life patient-reported management in these cases.

#### Group 2: Comparative GP Sample

To determine how representative COSAHC doctors’ patient management was, we compared their survey responses to those of a random sample of 39 GPs practicing within metropolitan Melbourne who had ordered routine blood tests (Full blood count and Urea and Electrolytes) in the same period from the same pathology provider.

Both groups of doctors were invited to return a survey tool by fax or complete over the telephone. The exclusion criteria were being on leave for more than one month at the time approached and/or no longer working at the practice. A sample size of 40 per group was recruited to provide 80% power (α = 0.05) to detect a 30% difference in antibiotic use between GPs.

The survey tool was developed to assess management protocols and swabbing practice in the context of a short case study of a patient with an axillary skin abscess. We also asked participants to provide information on any change to their practice over the previous three years.

#### Analysis

Descriptive statistics were applied, including computing differences between proportions and the associated *p*-values and 95% confidence intervals (CIs) using StataSE12. We report median and inter-quartile range (IQR) for all non-parametric data.

## Results

### COSAHC Participants Report of GP Management of Their Lesion

Abscess management and outcome information was obtained from the 66 index patients at the first COSAHC household visit. The susceptibilities of the organisms were known as the patients were recruited on the basis of a positive *S. aureus* MC&S result. The first household visit occurred at an average of 4 months from the onset of index infection. For the total COSAHC study, the ratio of MSSA:MRSA in the index patients’ infections was 154:137. Within this abscess subset, the ratio was 16:50. This indicates that in the setting of an abscess, MRSA is much more common than background rates in community onset infections. The average age of the abscess patients was 34.4 years (95% CI 29.3, 39.5), 48% were female (95% CI 37, 60), with an average household size of 2.7 (95% CI 2.25, 3.14). Nearly half (44% (95% CI 33, 56)) reported a history of previous skin abscesses. Abscess site varied (**Table 1**), however, leg/foot was the most common (over 30%). Torso (24%) ranked second for MRSA abscesses, while torso and arm/hand were equal second for MSSA abscesses (19%).

**Table 1:** Infection sites.

Site of infection	MSSA (*n* = 16) (%, CI)	MRSA (*n* = 50) (%, CI)
Leg/foot	31% (11, 59)	34% (21, 49)
Torso (front/back)	19% (4, 46)	24% (13, 38)
Arm/hand	19% (4, 46)	12% (5, 24)
Axilla	13% (2, 38)	12% (5, 24)
Head/neck	13% (2, 38)	8% (2, 19)
Groin	6% (0.2, 30)	10% (3, 22)

Abscess management by the GPs, as reported by COSAHC study patients, are shown in **Table 2** with index infections stratified by methicillin resistance. I&D was performed in 60.6% of cases, but never in isolation. Antibiotics were prescribed for 100% of cases, however, two patients reported not taking the antibiotics.

**Table 2:** Patient-reported GP abscess management by methicillin resistance (causal organism).

Treatment	MSSA (*n* = 16) (%, CI)	MRSA (*n* = 50) (%, CI)	Total (*n* = 66) (%, CI)
I&D and antibiotics	38% (15, 65)	36% (25, 53)	36% (25, 49)
I&D only	0%	0%	0%
Antibiotics only	25% (7, 52)	30% (18, 45)	29% (18, 41)
I&D, antibiotics and other^∗^	25% (7, 52)	24% (13, 38)	24% (15, 36)
Antibiotics and other^∗^	13% (2, 38)	10% (3, 22)	11% (4, 21)

*^∗^Other treatments include: abscess burst on its own, dressings, hospital admission, IV antibiotics, syringing, topical creams (non-antibiotic or unspecified), and surgical removal.*

Patient-reported infection resolution is shown in **Table 3**. Of those 40 patients who had I&D as part of their treatment, the infection resolved by the first home visit in 85% of cases, (95% CI 70, 94). Where I&D was not used (26) the infection was resolved in 73% of cases (95% CI 52, 88) by the first visit.

**Table 3:** Patient-reported infection resolution by first home visit (by management protocol).

	MSSA (*N* = 16)		MRSA (*N* = 50)		Total (*N* = 66)	
Treatment	Resolved	95% CI	Resolved	95% CI	Resolved	95% CI
I&D and antibiotics	5/6 (83%)	(36, 100)	15/18 (83%)	(59, 96)	20/24 (83%)	(63, 95)
I&D only	0 (0%)	–	0 (0%)	–	0 (0%)	–
Antibiotics only	3/4 (75%)	(19, 99)	12/15 (80%)	(52, 96)	15/19 (79%)	(54, 94)
I&D, antibiotics and other^∗^	3/4 (75%)	(19, 99)	11/12 (92%)	(62, 100)	14/16 (88%)	(62, 98)
Antibiotics and other^∗^	2/2 (100%)	(13, 99)	2/5 (40%)	(5, 85)	4/7 (57%)	(18, 90)

*^∗^Other treatments include: abscess burst on its own, dressings, hospital admission, IV antibiotics, syringing, topical creams (non-antibiotic or unspecified), and surgical removal.*

### Analysis of Antibiotic Use

**FIGURE 1:**
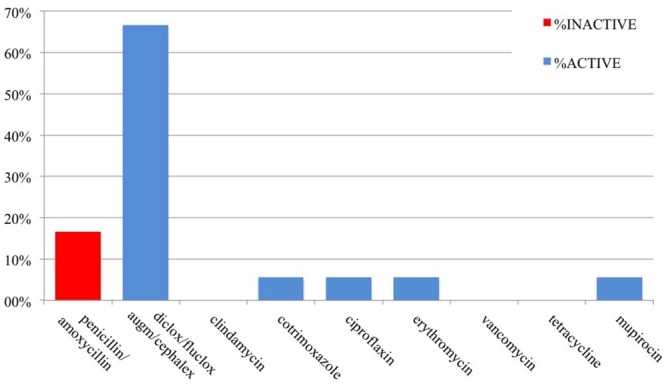
**Antibiotic susceptibility by prescription (*n* = 18) for MSSA infections**.

**FIGURE 2:**
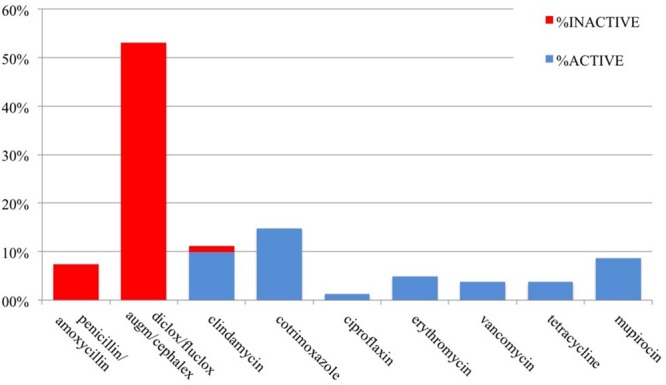
**Antibiotic susceptibility by prescription (*n* = 81) for MRSA infections**.

The antibiotics prescribed were not known for all patients, meaning the patient could recall the actual name of the antibiotic(s). However, the majority (85%) could remember the name of at least one of their prescribed antibiotics: 43 patients with MRSA and 13 patients with MSSA. We analyzed the antibiotic susceptibility profiles of the clinical isolates and the activity of the known antibiotics that were prescribed for these 56 patients (**Figures 1** and **2**). Antibiotics are described as *active* when the organism was susceptible to the prescribed antibiotic and *inactive* when the organism was resistant.

In total, over the course of their infections, there were 18 prescriptions of known antibiotics given to 13 patients with MSSA, of which three (16.7%) were inactive. Of the 81 known antibiotics prescribed for 43 patients with MRSA, 50 (62%) were inactive. Overall, approximately 53.5% of prescribed antibiotics were inactive: these included penicillin/amoxicillin for both MSSA and MRSA, and cephalexin, flucloxacillin, augmentin, or dicloxacillin for MRSA infections.

Prescriptions for MSSA infections were dominated by penicillin 16.7% and flucloxacillin, dicloxacillin, cephalexin, and augmentin (66.7%); However, augmentin and penicillin are not recommended first line of treatment for MSSA infections: augmentin because of its unnecessarily broad spectrum activity and penicillin/amoxicillin because only 5–10% of MSSA are susceptible (Turnidge et al., 2016).

### Antibiotic Changes and Repeat GP Visits

Of the 43 patients with MRSA infections who recalled at least one of their specific antibiotic(s), 35 (81%) were initially prescribed an inactive drug. Of these, 23% never received an active antibiotic, whilst the rest were subsequently prescribed active (58%) or unknown (19%) antibiotic(s) at follow-up visits. Three (23.1%) of 13 patients with MSSA who recalled the specific antibiotic(s) were also prescribed an inactive drug, with one subsequently prescribed an active antibiotic.

In our study, 56 patients were able to remember a total of 99 antibiotics (an additional 19 were remembered as a prescription but not by name). Thirty-seven patients (6 with MSSA and 31 with MRSA infections) were prescribed more than one antibiotic and, of those, thirteen (1 MSSA patients and 12 MRSA) were prescribed more than two antibiotics. In addition, eight were prescribed Mupirocin (site of use unspecified but all organisms susceptible). We presume that for every new antibiotic prescribed the patients required a repeat visit to their GP for assessment, although we do not know if the subsequent prescription changes were due to treatment failure or antibiotic change based on lab results showing resistance.

### GP Surveys of Abscess Management

One hundred and seventy GPs were approached and 81 completed surveys (48%; 41 COSAHC GPs, 39 comparison GP and one unlabeled form that could not be ascribed to a particular GP group). The response rate was higher in the COSAHC GP group (60% compared with 38%). The average number of years in practice of those participating was 23.1 years. No differences were found with treatment preferences between the two cohorts (**Table 4**).

**Table 4:** Self-reported management practices by doctor group.

Treatment	COSAHC GPs 41	Non-COSAHC	Total GPs (81)
	*n* (%^∗^)	GPs (39) *n* (%)	*n* (%)
I&D and antibiotics	29 (71%)	27 (69%)	57 (70%)
I&D	8 (20%)	7 (18%)	15 (19%)
Antibiotics only	3 (7%)	5 (13%)	8 (10%)
Other	1 (2%)	0 (0%)	1 (1%)

*^∗^All percentages are column percentages.*

Overall, GPs report ‘I&D and antibiotics’ as their preferred management of abscesses (70%), followed by ‘I&D only’ (19%) and ‘Antibiotics only’ (10%). Some specified I&D included additions; one would incise with review and another would prescribe antibiotic cream alongside. Other selections included ‘no initial treatment, but subsequent patient review’ (indicated as ‘other’ in **Table 4**).

### Decision to Swab Wound

When we stratified treatment choices according to the GPs decision to swab, we found abscess management did not differ significantly between the two surveyed doctor groups, with 70% of both groups reporting their preferred management protocols as ‘I&D and antibiotics.’ This indicates we have not introduced patient-management bias via the recruitment method used, other than including a greater proportion of patients treated by GPs who swab. More COSAHC doctors did indicate they would swab if the abscess was weeping or incised (76.9% vs. 56.4%; *p* = 0.049), but this was not shown to be associated with differences in patient management, so we pooled the two GP groups for further analyses.

Overall, a minority (29.9%) of GPs reported that they would routinely swab abscesses for culture. Doctors reported they would swab for definitive diagnostic purposes (68.8%) or for persistent abscesses (97.4%). About half of doctors (48.6%) report being influenced by abscess size and a perceived increased risk of systemic infection or spread to adjacent tissues. When abscess size was identified as important, thresholds of size varied from >1 cm to >3 cm. Swabbing practice was not related to treatment choice or the commencement of antibiotics.

The rise of MRSA in the community was identified as reason for changing practice (23%). Some doctors report they are now more likely to wait for results to prescribe antibiotics and choose to swab based on clinical site and severity of abscess.

### COSAHC Patient Management – GP-Reported Practice versus Actual Patient-Reported

Eight doctors of COSAHC patients with abscesses also completed the GP survey. Whilst the numbers are small, it does provide us the added opportunity of direct comparison between a patient’ reported abscess treatment and their doctor’s survey responses (**Table 5**).

**Table 5:** Community GP-reported management for abscesses versus patient-reported management.

GP survey response	Patients’ report of treatment by same GP
*Treatment of patient in case study*	*Treatment given for abscess*
Antibiotics	Antibiotics
Antibiotics	Antibiotics
I&D and Antibiotics	I&D and Antibiotics
I&D and Antibiotics	Self I&D, Antibiotics
I&D and Antibiotics	I&D attempt, penicillin injection, oral antibiotics
I&D and Antibiotics	I&D ×3, antibiotic injection, oral antibiotics
I&D and Antibiotics	Antibiotics (I&D of previous boil)
I&D and Antibiotics	Antibiotics

None of the eight responded with the recommended first line treatment for an abscess, regardless of size. Two reported that they would not perform I&D and would solely prescribe antibiotics, which corresponds to the patients’ report. The remaining six doctors who selected ‘I&D and Antibiotics’ in the survey varied in their actual clinical response – three out of six (50%) did not perform I&D and only prescribed antibiotics. Interestingly, two out of six (33%) administered an antibiotic injection as well as oral antibiotics. This is particularly surprising because there are no intramuscularly injectable antibiotics that are active against most *S. aureus* organisms.

## Discussion

We live in an era of rising antibiotic resistance. Conservation of antibiotics is of critical importance, and in response antibiotic stewardship programs are being progressively implemented. Antibiotic stewardship originated to address the problems of antibiotic overuse in hospitals, but is now being extended to the community as it is increasingly realized most antibiotics for human use are prescribed and consumed in the community.

Australian GP compliance with clinical guidelines for abscess treatment has not previously been researched. In the US the management of community-acquired abscesses have been investigated both in general practice and in hospital emergency departments (Odenholt et al., 2002; Daly et al., 2011; Forcade et al., 2011; Kemper et al., 2011; Parnes et al., 2011; May et al., 2012; Schmitz et al., 2013). Pre/post intervention studies in primary care have been conducted to evaluate abscess management. For example, the IRENE study looked at antibiotic prescription coverage – pre-intervention cephalosporins were frequently prescribed (43%), dropping to 18% of prescriptions post-intervention. In this cohort, 84% of patients received I&D and antibiotics pre-intervention. Post-intervention, 97% received antibiotics and I&D, but there was a demonstrated increase in MRSA susceptible antibiotics. The option to not prescribe antibiotics was discussed but did not translate into practice (Daly et al., 2011).

Similarly, in the SNOCAP-USA and DARTnet study (Parnes et al., 2011), procedure rates for I&D were initially low. A practical intervention had no significant effect on the number of I&D completed or for microbiological cultures, but again, did result in increased use of MRSA-susceptible antibiotics.

STARnet (Forcade et al., 2011) conducted a prospective community based observational study of suspected MRSA abscesses. Only 7% of this cohort had I&D alone, while 64% received both antibiotics and I&D. Twenty-eight percent of the cohort received only antibiotics with trimethoprim-sulfamethoxazole used frequently as monotherapy. These studies also demonstrate the reluctance to stop providing antibiotics alongside I&D, or at all, despite recommendations.

Hospital emergency department research in the US showed only 17–19% of abscesses were treated with I&D alone and treatment with antibiotics alone ranged from 4 to 17%, while a combination of I&D and antibiotics was the most common practice (66–79.9%; Moran et al., 2006; Talan et al., 2011; May et al., 2012; Schmitz et al., 2013). A quality improvement project (Kemper et al., 2011) was conducted amongst American pediatricians to develop an intervention to increase adherence to best practice of abscesses; 83% of those interviewed responded that they *could* perform I&D, however, 34% stated it was too time consuming to perform. The challenge exists then to emphasize the importance of I&D in treating patients with abscesses and encouraging best practice.

Our study focuses on community-onset abscess infections where MRSA is over-represented as a deliberate part of our sampling strategy. The data drawn from the prospective cohort of community-onset staphylococcal infections (COSAHC) identified via a private community-based pathology service, and the cross-sectional survey of doctors ordering tests through the same pathology service, has provided the opportunity to explore doctors’ attitudes and practices in the context of the changing epidemiology of *S. aureus* in an Australian urban community.

The antibiotic susceptibility of isolates for all the COSAHC participants were known and as this was the criteria for entry into the study. In the COSAHC study, we found that 85% of community-onset *S. aureus* infections were skin and soft tissue infections and in 66 the infection were skin abscesses and used in this study. In the management of the 66 participants with abscesses: 100% were prescribed antibiotics but only 60% had their abscess incised and drained. Contrary to this, a larger percentage of doctors (75%) surveyed indicated I&D with antibiotics was their management of choice for community-acquired abscesses. That is, more community GPs indicated that I&D with antibiotics was their management of choice than appeared to perform it.

Furthermore, where the antibiotic was known, the majority of patients with abscesses caused by MRSA (81%) were initially prescribed inactive antibiotics, compared with 23% of those with MSSA. Antibiotics were changed for 37 patients, resulting in nearly all MSSA (85%), but only 60.5% of MRSA infections ever being prescribed an active antibiotic. Overall, 53.5% of antibiotic choices were inactive against the targeted pathogen.

We have previously estimated that about 8% of infections caused by *S. aureus* in the community of metropolitan Melbourne are MRSA (Bennett et al., 2014), but this is likely to be much higher for abscesses based on our findings here. Given the proportion of COSAHC community-onset infections that are abscesses (66/291, 23%), and the high rate due to MRSA infections (78% compared with 47% of all infections among the frequency-matched COSAHC index cases), we estimate the prevalence of MRSA infections might be as high as 24% (three times higher) in community-onset infections presenting as abscesses in metropolitan Melbourne 2010–2012.

From our survey we found that only 30% of community GPs swab for MC&S. Therefore, many doctors would be unaware of the proportion of antibiotics prescribed that were ineffective and they are unlikely to modify their prescribing practice. According to both contemporary and Australian guidelines, almost all of the prescriptions for uncomplicated abscesses could be considered unnecessary. In support of these guidelines, our study showed there was little difference in infection resolution whether the antibiotics were active or not. This was true for ‘I&D + antibiotics’ and ‘antibiotics’ alone (*p* = 0.71), whether the antibiotics were active (*p* = 1.0) or inactive (*p* = 0.84). However, there was a trend to greater resolution at first household visit for those patients who had I&D performed, with 85% of infections resolved (95% CI 70, 94) compared with 73.1% (95% CI 52.2, 88.4) of patients who did not have I&D performed. A 2007 literature review (Hankin and Everett, 2007) also showed that those patients treated with I&D alone had the same rate of resolution as those with I&D and antibiotics.

If an antibiotic is prescribed it is usually at first presentation. The swab will not help in the initial antibiotic prescription but a MC&S may be useful in the case of treatment failure and MRSA surveillance. If I&D is performed correctly, our findings support guidelines and no antibiotics are needed and can be stopped. Patients’ expectations regarding treatment is a challenge for GPs. Patient understanding of best practice can be narrow and limited to their own situation. There is a need to increase the health literacy of patients and to make them aware of the population wider risks associated with antibiotic over prescription.

Although I&D was consistently reported by GPs to be their preferred treatment option for uncomplicated abscesses, the COSAHC study observed only 60% of patients with abscesses were treated with I&D in practice. Furthermore, I&D was always accompanied by antibiotics. This suggests that whilst there may be an understanding of the importance of I&D, there may be a lack of confidence in performing the procedure without antibiotic cover.

Given the number of antibiotic changes (37 patients requiring additional antibiotics) and therefore the presumed increased number of visits to the GP, it is clear that skin abscesses are difficult to treat and many different antibiotics are used.

Our study has a number of limitations. The response rate of 48% in the GP survey has the potential for sampling bias. However, we believe that any selection bias that may have been introduced would act to favor doctors who are more aware of community MRSA and therefore more keen to participate. Therefore, it is possible that these results underestimate the actual deviation from recommended practice in the wider GP community. If this were the case, then the true situation regarding GP practices may be even more removed from therapeutic practice guidelines than captured here. It would be useful to conduct a larger study to more comprehensively examine GP practice, and gain more detailed insight into GPs’ ability to perform I&D correctly. Incision type, use of pain control, irrigation, wound cultures, and packing, would inform targeted strategies (Forcade et al., 2011; Centers for Disease Control and Prevention [CDC], 2013).

## Conclusion

Our findings demonstrate many GPs are not following guideline recommended practice when it comes to treating patients with staphylococcal skin abscesses. Our findings support the recommendations in the current antibiotic guidelines that would act to curb this trend (Antibiotic Expert Groups, 2014). In particular:

(1) Antibiotics not indicated unless spreading cellulitis with systemic symptoms.
(2) Perform I&D correctly on all abscesses, even if antibiotic therapy is considered.
(3) Modify therapy based on clinical response to initial therapy and the results of cultures and susceptibility testing. If *S. aureus* is isolated, and lesion is responding to drainage, stop any antibiotics, inactive and active. If antibiotics are necessary: treatment for 5 days is generally sufficient, but a longer duration of therapy may be required for patients who are slow to respond or have a more severe infection.

Overall, the doctors we surveyed demonstrated awareness of the changing epidemiology of *S. aureus* infections in Melbourne. This study provides a basis for developing new programs to assist GPs to better understand and reduce their use of antibiotics, and potentially the number of return patient visits. There is also vital need to improve confidence and proficiency or surgical techniques such as I&D, which are more effective than antibiotics in such cases.

## Author Contributions

CP initiated this work, lead the study design and ethics approval, and managed the recruitment and oversaw the data analysis, and instigated the writing of this manuscript. GW was responsible for managing the partnership with Dorevitch laboratory and GP recruitment and contributed to the design of the survey tool, interpretation of data, and manuscript preparation. EB assisted in data analysis and preparing the figures for this paper. GC was responsible for the *S. aureus* laboratory typing and determination of resistance, and contributed to these elements of the paper. PJ also contributed to the development of the survey tool and to the writing of the manuscript, especially the clinical relevance aspects in the discussion and conclusions of this manuscript. CB developed the concept for this research with CP, lead the COSAHC study that contributed data to this work, oversaw the data analysis and data reporting processes, and contributed to the initial writing and then polishing of this manuscript.

## Conflict of Interest Statement

The authors declare that the research was conducted in the absence of any commercial or financial relationships that could be construed as a potential conflict of interest.

## References

[B1] Antibiotic Expert Groups (2014). *Therapeutic Guidelines: Antibiotic, Version 15*. Melbourne, VIC: Therapeutic Guidelines Limited.

[B2] Bennett C. M. Coombs G. W. Wood G. M. Howden B. P. Johnson L. E. A. White D. (2014). Community-onset *Staphylococcus aureus* infections presenting to general practices in South-eastern Australia. *Epidemiol. Infect.* 142 501–511. 10.1017/S0950268813001581 23866772 PMC9151107

[B3] CDC AMA IDSA (2007). *CDC Outpatient Management of MRSA Skin and Soft Tissues Infections in the Era of Community-Associated MRSA [Internet].* Atlanta, GA: Centers for Disease Control and Prevention.

[B4] Centers for Disease Control and Prevention [CDC] (2013). *Treating MRSA Skin and Soft Tissue Infections in Outpatient Settings [Internet].* Atlanta, GA: Centers for Disease Control and Prevention.

[B5] Coombs G. W. Daley D. A. Pearson J. C. Nimmo G. R. Collignon P. J. McLaws M.-L. (2014). Community-onset *Staphylococcus aureus* surveillance Programme Annual Report 2012. *Commun. Dis. Intell. Q. Rep.* 38 E59–E69. 25409357 10.33321/cdi.2014.38.12

[B6] Daly J. M. Levy B. T. Ely J. W. Swanson K. Bergus G. R. Jogerst G. J. (2011). Management of skin and soft tissue infections in community practice before and after implementing a “best practice” approach: an iowa Research Network (IRENE) Intervention Study. *J. Am. Board Fam. Med.* 24 524–533. 10.3122/jabfm.2011.05.110017 21900435

[B7] del Giudice P. Blanc V. de Rougemont A. Bes M. Lina G. Hubiche T. (2009). Primary skin abscesses are mainly caused by Panton-Valentine Leukocidin-positive *Staphylococcus aureus* strains. *Dermatology* 219 299–302. 10.1159/000232391 19648730

[B8] Dermatology Expert Groups (2009). *Therapeutic Guidelines: Dermatology, Version 3*. Melbourne, VIC: Therapeutic Guidelines Limited.

[B9] Forcade N. A. Parchman M. I. Jorgensen J. H. Du L. C. Nyren N. R. Trevino L. B. (2011). Prevalence, severity, and treatment of community-acquired methicillin-resistant *Staphylococcus aureus* (CA-MRSA) skin and soft tissue infections in 10 medical clinics in texas: a South Texas Ambulatory Research Network (STARNet) study. *J. Am. Board Fam. Med.* 24 543–550. 10.3122/jabfm.2011.05.110073 21900437 PMC3258020

[B10] Hankin A. Everett W. W. (2007). Are antibiotics necessary after incision and drainage of a cutaneous abscess? *Ann. Emerg. Med.* 50 49–51. 17577944 10.1016/j.annemergmed.2007.01.018

[B11] Jahamy H. Ganga R. Al Raiy B. Shemes S. Nagappan V. Sharma M. (2008). *Staphylococcus aureus* skin/soft-tissue infections: the impact of SCCmec type and Panton-Valentine leucocidin. *Scand. J. Infect. Dis.* 40 601–606. 10.1080/00365540701877312 18979597

[B12] Kemper A. R. Dolor R. J. Gowler V. G. Jr. (2011). Management of skin abscesses by primary care pediatricians. *Clin. Pediatr.* 50 525–528. 10.1177/0009922810394837 21262755

[B13] Liu C. Bayer A. Cosgrove S. E. Daum R. S. Fridkin S. K. Gorwitz R. J. (2011). Clinical practice guidelines by the infectious diseases society of america for the treatment of methicillin-resistant *Staphylococcus aureus*. *Clin. Infect. Dis.* 52 285–292. 10.1093/cid/ciq146 21217178

[B14] Lowy F. D. Sexton D. J. Baron E. L. (2008). *Treatment of Skin and Soft Tissue Infections Due to Methicillin-Resistant Staphylococcus aureus in Adults.* Waltham, MA: UpToDate.

[B15] May L. Harter K. Yadav K. Strauss R. Abualenain J. Keim A. (2012). Practice patterns and management strategies for purulent skin and soft-tissue infections in an urban academic ED. *Am. J. Emerg. Med.* 30 302–310. 10.1016/j.ajem.2010.11.033 21277138

[B16] Moran G. Krishnadasan A. Gorwitz R. Fosheim G. E. McDougal L. K. Carey R. B. (2006). Methicillin-resistant *S. aureus* infections among patients in the emergency department. *New Engl. J. Med.* 355 666–674. 10.1056/NEJMoa055356 16914702

[B17] Moran G. J. Abrahamian F. M. LoVecchio F. Talan D. A. (2013). Acute bacterial skin infections: developments since the 2005 Infectious Diseases Society of America (IDSA) guidelines. *J. Emerg. Med.* 44 e397–e412. 10.1016/j.jemermed.2012.11.050 23466022

[B18] Odenholt I. Bylander-Groth A. Fridmodt-Moller N. Rokstad K. S. Molstad S. (2002). Differences in antibiotic prescribing patterns between general practitioners in Scandinavia: a questionnaire study. *Scand. J. Infect. Dis.* 34 602–609. 12238578 10.1080/00365540210147624

[B19] Parnes B. Fernald D. Coombs L. DeAlleaume L. Brandt E. Webster B. (2011). Improving the management of skin and soft tissue infections in primary care: a report from State Networks of Colorado Ambulatory Practices and Partners (SNOCAP-USA) and the Distributed Ambulatory Research in Therapeutics Network (DARTNet). *J. Am. Board Fam. Med.* 24 534–542. 10.3122/jabfm.2011.05.110018 21900436

[B20] Schmitz G. Goodwin T. Singer A. Kessler C. S. Bruner D. Larrabee H. (2013). The treatment of cutaneous abscesses: comparison of emergency medicine providers’ practice patterns. *West. J. Emerg. Med.* 14 23–28. 10.5811/westjem.2011.9.6856 23447753 PMC3582519

[B21] Talan D. A. Krishnadasan A. Gorwitz R. J. Fosheim G. E. Limbago B. Albrecht V. (2011). Comparison of *Staphylococcus aureus* from skin and soft-tissue infections in US emergency department patients, 2004 and 2008. *Clin. Infect. Dis.* 53 144–149. 10.1093/cid/cir308 21690621

[B22] Turnidge J. Rao N. Chang F.-Y. Fowler V. G. Kellie S. M. Arnold S. (2016). “*Staphylococcus aureus*: susceptibility in vitro and in vivo,” in *Antimicrobial Therapy and Vaccines: Microbes* Vol. 1. Available at: http://www.antimicrobe.org/ (accessed April 26, 2016).

